# Primary Gastric Squamous Cell Carcinoma

**DOI:** 10.7759/cureus.2389

**Published:** 2018-03-28

**Authors:** Patricia Guzman Rojas, Jignesh Parikh, Priya Vishnubhotla, Juan J Oharriz

**Affiliations:** 1 Internal Medicine, UCF College of Medicine; 2 Pathology, Orlando VA Medical Center; 3 Medicine, Hematology-Oncology, Orlando VA Medical Center; 4 Gastroenterology, Orlando VA Medical Center

**Keywords:** gastric cancer, squamous cell cancer

## Abstract

Primary gastric squamous cell carcinoma (PGSCC) is an extremely rare cause of gastric malignancy.

We present a 66-year-old man with a past medical history of stage I left palpebral marginal zone lymphoma status post radiation. The patient was complaining of a two-year history of bloating and early satiety. An upper endoscopy was performed, showing a 2.5 cm polypoid lesion at proximal corpus; however, the cardia and esophagus were normal. Biopsies were positive for gastric squamous cell carcinoma. He underwent partial gastrectomy and was referred to oncology for treatment.

In 2011, the Japanese Gastric Cancer Association proposed diagnostic criteria for the diagnosis of PGSCC. The clinical presentation of this malignancy does not differentiate from that of other types of gastric tumors. We report this case to highlight squamous cell carcinoma as a cause of primary gastric cancer. Gastroenterologists should be aware of this entity to facilitate prompt referral to specialized centers, where surgical resection can be done.

## Introduction

It is well known that adenocarcinoma accounts for most of the causes (approximately 95%) of gastric malignancies [1]. There are several different histological types of gastric cancer (Table 1) [2]; however, primary gastric squamous cell carcinoma (PGSCC) is an extremely rare malignancy that shows a frequency of 0.04%-0.07% among all gastric cancers, according to what is described by Straus et al. [3].

**Table 1:** WHO classification of tumors of the digestive system

World Health Organization (2010): Classification of Tumours of the Digestive System	
-Papillary adenocarcinoma	-Carcinosarcoma
-Tubular adenocarcinoma	-Parietal cell carcinoma
-Mucinous adenocarcinoma	-Malignant rhabdoid tumor
-Signet-ring cell carcinoma	-Mucoepidermoid carcinoma
-And other poorly cohesive carcinoma	-Paneth cell carcinoma
-Mixed carcinoma	-Undifferentiated carcinoma
-Adenosquamous carcinoma	-Mixed adeno-neuroendocrine carcinoma
-Squamous cell carcinoma	-Endodermal sinus tumor
-Hepatoid adenocarcinoma	-Embryonal carcinoma
-Carcinoma with lymphoid stroma	-Pure gastric yolk sac tumor
-Choriocarcinoma	-Oncocytic adenocarcinoma

## Case presentation

**Figure 1:**
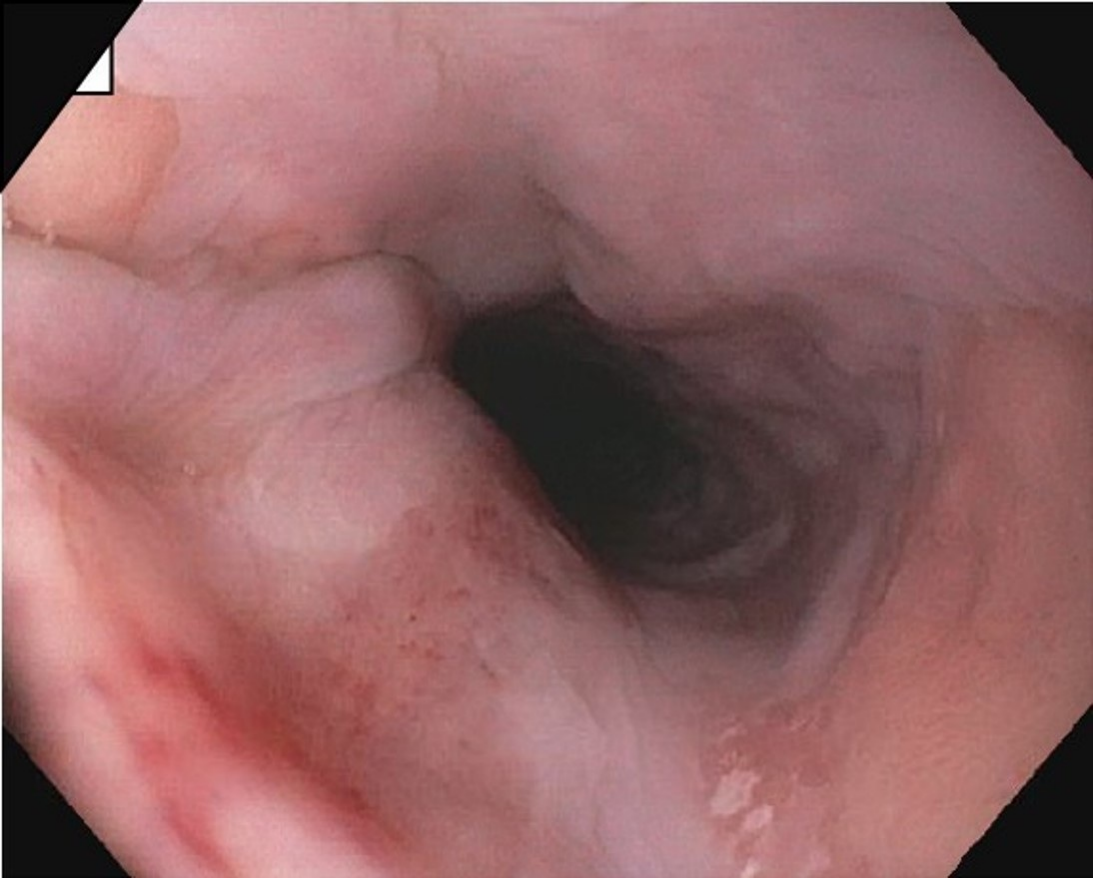
Esophageal mucosa without any masses/tumors. Biopsies taken were normal. EGD: Esophagogastroduodenoscopy

**Figure 2:**
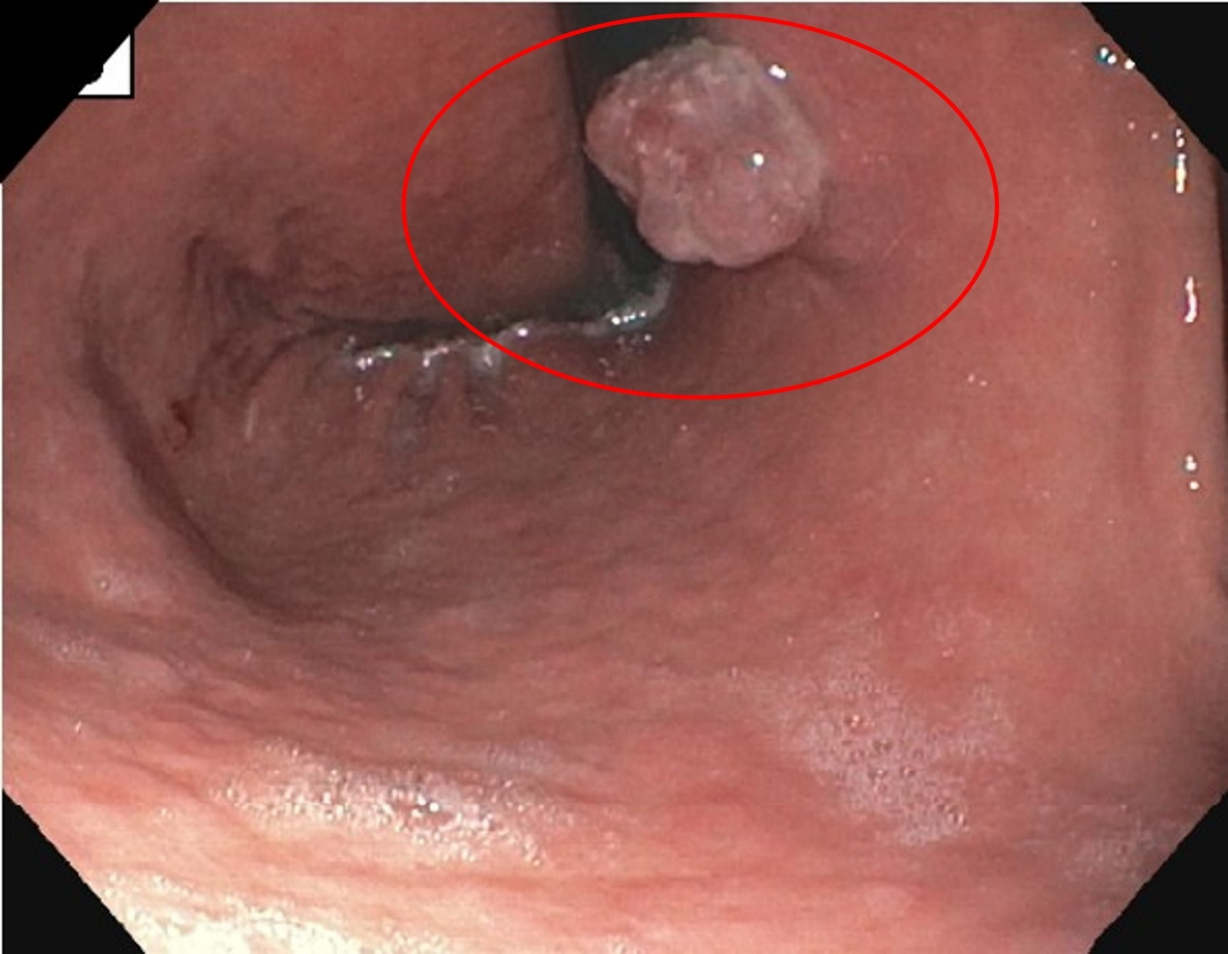
A 2.5 cm polypoid lesion with a wide base at proximal corpus toward lesser curvature. EGD: Esophagogastroduodenoscopy

**Figure 3:**
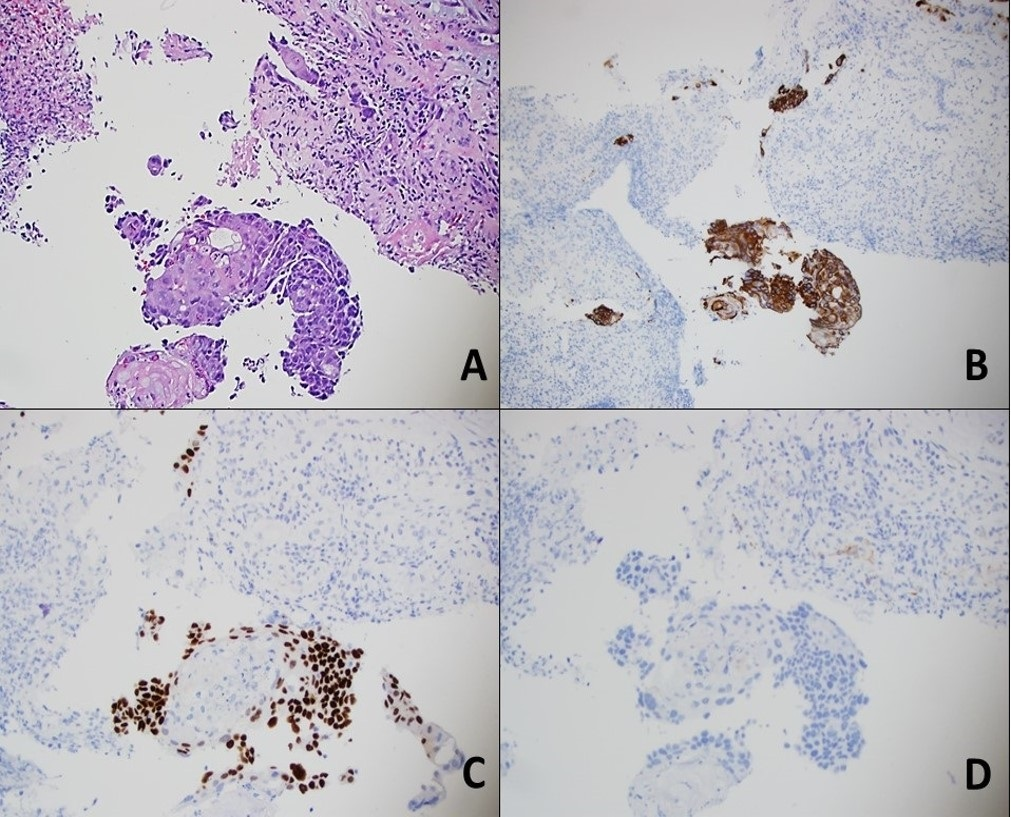
Histopathologic exam of biopsy from stomach body shows carcinoma with squamous differentiation (Panel A). The tumor cells are positive for CK 5/6 (Panel B) and p63 (Panel C) and negative for CK 7 (Panel D). This immunophenotype is consistent with squamous cell carcinoma.

A 66-year-old man presented to the gastroenterology clinic complaining of a two-year history of bloating and early satiety. He had a past medical history of diabetes mellitus, coronary artery disease status post stent placement, hypertension, and stage I left palpebral marginal zone lymphoma status post radiation. He denied any prior history of smoking, dysphagia, hematemesis, melena, hematochezia, rectal bleeding, or unintentional weight loss.Due to the reported symptoms, an esophagogastroduodenoscopy (EGD) was performed, showing a normal esophagus (Figure 1) and a 2.5 cm polypoid lesion with a wide base at the proximal corpus toward the lesser curvature and distant to the gastric cardia (Figure 2). A biopsy of the polypoid lesion showed gastric squamous cell carcinoma (Figure 3), whereas the gastroesophageal junction biopsy showed normal mucosa. Histopathology elucidated tumor cells positive for cytokeratin 5/6 (CK 5/6) and p63 and negative for CK7, CK20 (Figure 3, panels A-D).

Based on the histopathology results, the patient underwent an endoscopic ultrasound (EUS) and attempted endoscopic mucosal resection (EMR). The EUS showed evidence of a hypoechoic irregular mass at the body of the stomach with sonographic evidence suggesting invasion into the submucosa (layer three of five) and abutting the muscularis propria. Per sonographic criteria, this malignancy was categorized as T2 N0 Mx. An EMR was attempted for a snare mucosal resection and was unsuccessful to obtain tissue for staining.

A positron emission tomography (PET) scan demonstrated a 24-mm markedly hypermetabolic lesion in the gastric body, less curvature, and no other lesions in the body. Based on the results of the PET scan and immunohistochemistry, this patient had no evidence of malignancy elsewhere, further confirming that this tumor is gastric in origin; more specifically, a primary gastric squamous cell carcinoma. The patient was referred to surgical oncology and underwent a proximal gastrectomy. Referral to medical oncology was also arranged for initiating 5-fluorouracil chemotherapy.

## Discussion

PGSCC is an uncommon entity and, currently, there are only 100 cases reported in the worldwide literature [1,4]. Diagnostic criteria for PGSCC were first described in 1967 [5]. To meet the diagnostic criteria, three features are required: a) the tumor should not be located at the cardia, b) the tumor should not extend into the esophagus, and c) the patient should not have evidence of squamous cell carcinoma (SCC) in any other part of the body. In 2011, the Japanese Gastric Cancer Association proposed updated criteria [6] that comprise the following: a) all tumor cells must be SCC cells without any gland cancer cells and b) SCC must originate in the gastric mucosa.

The exact origin of this tumor is not known; however, several theories exist [3,7]. These include: (1) “totipotential cell” that can give rise to any cell type, (2) squamous metaplasia in a pre-existing non-neoplastic glandular epithelium, 3) ectopic squamous cells nests, 4) squamous differentiation of adenocarcinoma, 5) vascular endothelium, and 6) Epstein-Barr virus or human papillomavirus [4].

A recent retrospective analysis, published in 2016, reviewed 21 patients with PGSCC. Similar to our patient, they found a male predominance (with a male:female ratio of 6:1) [8]. Furthermore, in the Wakabayashi et al. study, half of the patients presented with a T4 stage tumor; different from our patient, who presented with a T2 stage tumor [9].

The clinical presentation of this malignancy does not differ from other types of gastric tumors, as patients can have nonspecific abdominal pain, nausea, vomiting, melena, weight loss, early satiety, and bloating [8]. 

Currently, the main therapeutic approach is surgery involving gastrectomy and lymph node resection. Also, adjuvant chemotherapy is given, consisting of 5-fluorouracil-based regimens, which are the typical treatment for gastric adenocarcinoma. Other alternatives used are platin and taxane-based regimens. These include docetaxel + oxaliplatin/cisplatin + fluorouracil (DOF), fluorouracil + oxaliplatin + calcium folinate (FOLFOX), capecitabine + oxaliplatin (XELOX), docetaxel + capecitabine, cisplatin + fluorouracil/campitabine/S-1, docetaxel + cisplatin/oxaliplatin (TP), gemcitabine + fluorouracil (GF) and pirabucin + fluorouracil [10]. It is important to mention that some reports have found neoadjuvant chemotherapy for PGSCC to be effective [4]; however, there is no definitive role according to the available data [8].

A new retrospective study published by Meng et al. showed a poorer prognosis in patients with advanced gastric SCC, compared with adenocarcinoma of the stomach, with a median survival of seven months [10]. This is due to the advanced stage at the time of diagnosis [8-9].

## Conclusions

This case intends to highlight squamous cell carcinoma as a cause of primary gastric cancer. Gastroenterologists should be aware of this entity to facilitate prompt referral to specialized centers that offer advanced diagnostic procedures and a full repertoire of investigational and non-investigational agents/protocols for treatment.
